# Public health advocacy strategies to influence policy agendas: lessons from a narrative review of success in trade policy

**DOI:** 10.1186/s12992-023-00960-7

**Published:** 2023-08-23

**Authors:** Belinda Townsend, Brigitte Frances Tenni, Sharni Goldman, Deborah Gleeson

**Affiliations:** 1 Australian Research Centre for Health Equity, School of Regulation and Global Governance, Australian National University, Canberra, Australia; 2 School of Psychology and Public Health, La Trobe University, Bundoora, VIC 3086 Australia; 3 Nossal Institute for Global Health, The School of Population and Global Health, The University of Melbourne, Carlton, VIC 3010 Australia; 4 Australian Research Centre for Health Equity, School of Regulation and Global Governance, Australian National University, Canberra, Australia

**Keywords:** Trade, Access to medicines, Social determinants of health, Trade agreements, Trade policy, Health policy, Health equity, Public health, Commercial determinants of health

## Abstract

**Background:**

Despite accumulating evidence of the implications of trade policy for public health, trade and health sectors continue to operate largely in silos. Numerous barriers to advancing health have been identified, including the dominance of a neoliberal paradigm, powerful private sector interests, and constraints associated with policymaking processes. Scholars and policy actors have recommended improved governance practices for trade policy, including: greater transparency and accountability; intersectoral collaboration; the use of health impact assessments; South-South networking; and mechanisms for civil society participation. These policy prescriptions have been generated from specific cases, such as the World Trade Organization’s Doha Declaration on TRIPS and Public Health or specific instances of trade-related policymaking at the national level. There has not yet been a comprehensive analysis of what enables the elevation of health goals on trade policy agendas. This narrative review seeks to address this gap by collating and analysing known studies across different levels of policymaking and different health issues.

**Results:**

Sixty-five studies met the inclusion criteria and were included in the review. Health issues that received attention on trade policy agendas included: access to medicines, food nutrition and food security, tobacco control, non-communicable diseases, access to knowledge, and asbestos harm. This has occurred in instances of domestic and regional policymaking, and in bilateral, regional and global trade negotiations, as well as in trade disputes and challenges. We identified four enabling conditions for elevation of health in trade-related policymaking: favourable media attention; leadership by trade and health ministers; public support; and political party support. We identified six strategies successfully used by advocates to influence these conditions: using and translating multiple forms of evidence, acting in coalitions, strategic framing, leveraging exogenous factors, legal strategy, and shifting forums.

**Conclusion:**

The analysis demonstrates that while technical evidence is important, political strategy is necessary for elevating health on trade agendas. The analysis provides lessons that can be explored in the wider commercial determinants of health where economic and health interests often collide.

**Supplementary Information:**

The online version contains supplementary material available at 10.1186/s12992-023-00960-7.

## Introduction

Noncommunicable diseases (NCDs) such as cardiovascular disease, diabetes and lung cancer present an ‘invisible health epidemic’ described by the World Health Organization (WHO) as ‘one of the major challenges for development in the 21st century’ [1]. The economic, social and health costs from rising NCDs are significant. Yet, the role of trade and investment agreements in facilitating greater access to NCD risk factors such as tobacco, harmful use of alcohol and ultra-processed foods is often overlooked. Governments have committed to several high level international NCD action plans while at the same time expanding their trade and investment agreements, facilitating greater access to cheap health-harming commodities and introducing new constraints on public health regulation [2–7].

In response, public health experts have provided important technical analysis to inform the drafting of trade agreements and trade-related policies in ways that can mitigate impacts on health [8, 9]. Indeed, trade can have positive impacts for health, depending on the specifics. Yet, despite accumulating evidence of the potential negative or positive implications for public health, trade and health sectors continue to operate largely in silos [10]. Numerous barriers to advancing health have been identified, including the dominance of a neoliberal paradigm – by which we refer to ‘the new political, economic and social arrangements within society that emphasize market relations [and] re-tasking the role of the state,’ [11], powerful private sector interests, and constraining policymaking processes [10, 12–21]. The dominance of a neoliberal paradigm in many countries provides government officials and industry officials with a shared framing and common objective to promote exports and market access [22, 23]. Transnational corporations that produce and/or market commodities harmful to health influence trade agendas through lobbying, framing, and access to institutional processes [19]. Many governments have faced trade challenges to their health policies at various World Trade Organization (WTO) committees, including those related to alcohol, nutrition and tobacco [24–29]. Jarman [30] and van Schalkayk et al. [21] identify key governance challenges regarding transparency, accountability, participation, integrity and capacity.

Scholars and policy actors have recommended improved governance practices for trade policymaking including: greater transparency and accountability, the representation of health officials on government trade delegations, the use of health impact assessments, South-South networking, and mechanisms for civil society participation [9, 23, 31–35]. These policy prescriptions have been primarily generated from experience with, or studies of, specific instances, such as the WTO Doha Declaration on Trade-Related Aspects of Intellectual Property Rights (TRIPS) and Public Health [36, 37], or specific instances of trade-related policymaking at the national level [23, 25, 31, 38–40]. There has not yet been a comprehensive analysis of the strategies and conditions that have enabled the elevation of health in trade-related policymaking across different contexts given these governance challenges. This narrative review seeks to address this gap by collating and analysing studies across different levels of policymaking and different health issues. In doing so, it aims to identify what has worked for policy actors seeking to advance health in trade-related policy agendas. This aim is important both for policy scholars seeking to understand the mechanisms for change, and for public health advocates wanting to advance health goals.

## Method

A narrative review was selected for the review method [41] because of the interdisciplinary nature of the topic and the qualitative focus of much of the literature. This involved a systematic search for relevant scholarly literature across a range of disciplines including political science, international relations, public health, economics, and law, followed by analysis and thematic synthesis of the results.

### Search process

A systematic search of relevant peer-reviewed scholarly literature was conducted in November 2019 and then updated in November 2022 using four comprehensive and relevant scholarly databases: Web of Science, Scopus, Pubmed and Global Health. Search terms were chosen for three concept categories: social determinants of health, governance and political factors, and trade policy terms (see Table 1). Social determinants of health terms were chosen by reviewing literature on the health impacts of trade agreements and associated literature on the social determinants of health [42, 43]. Governance and political factors and trade terms were identified from the authors’ review of known publications on trade governance and health and associated literature [43]. The search string was revised in consultation with a librarian and through preliminary searches. Peer-reviewed articles, book chapters, and books were included in the search.

**Table 1:** Search terms

Category	Search terms
Social determinants of health	social determinants of health, cross border healthcare, cross-border healthcare, intellectual property, sustainable development, health*, health policy, health services, access to medicines, health in all policies, food*, nutrition, diet-related health, food security, non-communicable disease, ncd, health diplomacy
Governance and political factors	advocacy, agenda*, attention, framing, priorit*, commitment, enable, constrain, influenc*, negotiat*, policy-mak*, govern*, polic*, politic*, problemati?ation, consult*
Trade policy terms	international trade, trade policy, trade liberalisation, trade liberalization, trade governance, trade agreement*,world trade organisation, world trade organization, TRIPS agreement, TPP, NAFTA, RCEP

Results from the database search (18,604 records) were collated in an Endnote library and then uploaded to the Covidence platform, where duplicates were excluded. Due to the authors’ native language constraints, only English language articles were included. Articles were included if they provided an empirical study of strategies and/or conditions which led to the elevation of health goals in trade-related policymaking, whether that be at the national, regional and/or global level. We defined trade-related policymaking as including: national, regional and multilateral trade policy; bilateral, regional and multilateral trade negotiations; studies of trade challenges and disputes; and papers that examined the conditions enabling the elevation of health goals in trade-related policy implementation. Articles were excluded if 1) the paper was not in English, 2), it was unrelated to trade policy, or 3) the analysis did not identify strategies or conditions that enabled elevation of a health goal (i.e. studies that were descriptive, prospective, or focused on technical details in the absence of an account of what happened and why). BT, BFT and DG screened records in Covidence by title and abstract and then screened the same four full-text studies to test the inclusion and exclusion criteria. To ensure inter-assessor reliability, BT, BFT and DG each screened a 25% sample of the full-texts studies, with disagreements identified and resolved through discussion. BT then screened all full-text studies. Of the full-text studies read, 243 were excluded using the inclusion and exclusion criteria, resulting in a total of 60 included studies. The reference lists of included articles were also reviewed for relevant articles that had not been captured by the search, which were added manually for screening, adding an additional 5 studies. A total of sixty-five documents were included for analysis, comprising fifty-seven journal articles, six book chapters and two books.

**Fig. 1:**
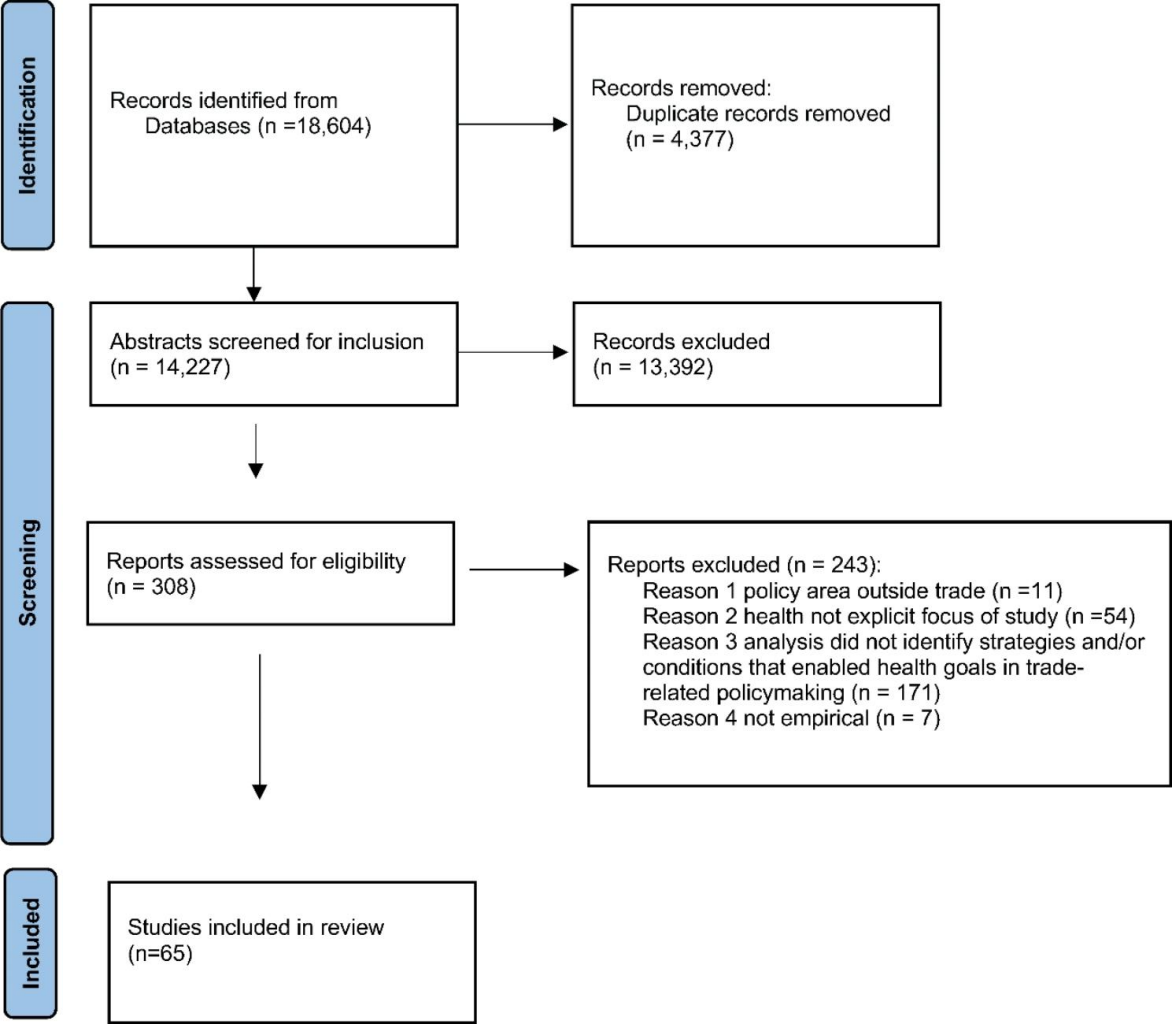
Flow diagram of screening results

Quality appraisal was undertaken during the full-text stage, adopting best practice guidance on appropriateness of study design, evidence of data sources, a clear statement of findings and justifiable conclusions [41]. A flow diagram of the screening results is provided in Fig. 1.

### Analysis

Our approach is influenced by a modern realist epistemology, in which we use theory to identify how social structures constrain and enable outcomes [44]. We drew on institutional theory to assist with thematic coding and synthesis of the included studies. Institutional theory positions interests, ideas, and institutions as variables in understanding policymaking [45]. Interests refer to the agendas of governance actors, including societal groups, elected officials, civil servants, researchers, industry actors and policy entrepreneurs. Actors identified in the trade governance literature who have contributed to advancing health goals in trade policy, for example, include low and middle-income country (LMIC) governments and civil society organisations [12, 46, 47]. Ideas refer to the understandings actors bring to the issue, shaped by their values, beliefs and ideologies, including the role of framing in generating support (or opposition) to the issue [45]. Successful framing occurs when advocates secure attention to their desired problem and solution through convincing arguments and narratives [12, 37]. Institutions are defined as the formal and informal rules of the game. Institutions include government structures and policy legacies which shape policymaking in ways that favour some interests or ideas over others. The formal and informal policy processes of trade negotiations, for example, can enable or constrain health advocacy [10, 13, 23], as can the structures of government coordination [38, 40]. We added a further category for ‘issue characteristics’ - drawing on Shiffman & Smith’s [48] framework of political prioritisation in global health, which has been applied in studies of trade and health [23, 49] - to capture the potential influence of particular characteristics of health issues, such as the strength of the evidence base.

All studies were coded in NVivo qualitative software using a coding scheme developed from the theoretical framework (interests, ideas, institutions and issue characteristics). Coding was both deductive, guided by the theoretical framework, and inductive, with new codes emerging from the analysis of the studies and concepts integrated and added through the analysis. The data were then organised into a final set of key themes. In NVivo, we conducted a matrix-coding query for themes for each category of trade policymaking. In doing so, we identified a set of factors that were reported to be influential across the categories of trade-policymaking that were outside the control of policy actors (i.e. the ‘conditions’). We identified themes of strategies that policy actors used to influence those conditions (the ‘strategies). These are detailed in the results. Data on study characteristics were also recorded by SG, BFT and BT in Microsoft Excel, including the author(s), title, year of publication, study type, level of analysis (national, regional, global), country/countries studied, public health issue, and type of trade-related policy (See Supplementary Table).

## Results

**Fig. 2:**
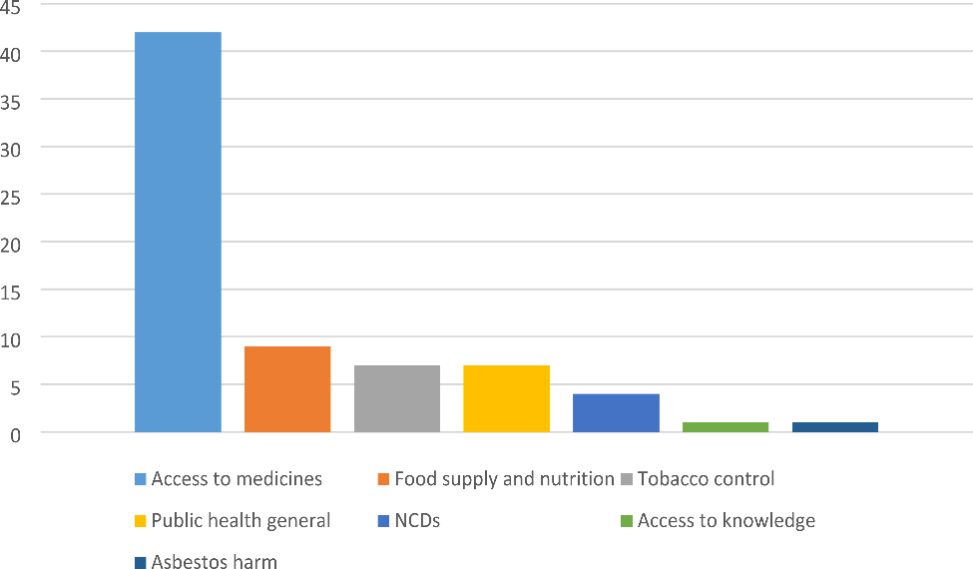
Public health issue in focus

Sixty-five studies (from 18,604 studies screened) identified conditions and/or strategies that enabled the elevation of a health goal in trade-related policymaking. Access to medicines was the most studied public health issue (n = 42), followed by food (nutrition and/or food security) (n = 9), tobacco control (n = 7), non-communicable disease risk factors (n = 4), access to knowledge (n = 1)^1^, and asbestos harm (n = 1). Papers about health in general and not explicitly linked to specific issues were coded as ‘public health general’ (n = 7) (See Fig. 2).

### Trade-related policymaking

We grouped studies of trade-related policymaking into seven sub-categories: trade negotiations between two or more countries (n = 28), national government positions taken in trade negotiations (n = 11), trade challenges and disputes (n = 10), national level trade policymaking (n = 6), national policy implementation (n = 4), ‘trade-proofing’ health policy (n = 4) and national level trade bans (n = 2).

### Level of policymaking

**Fig. 3:**
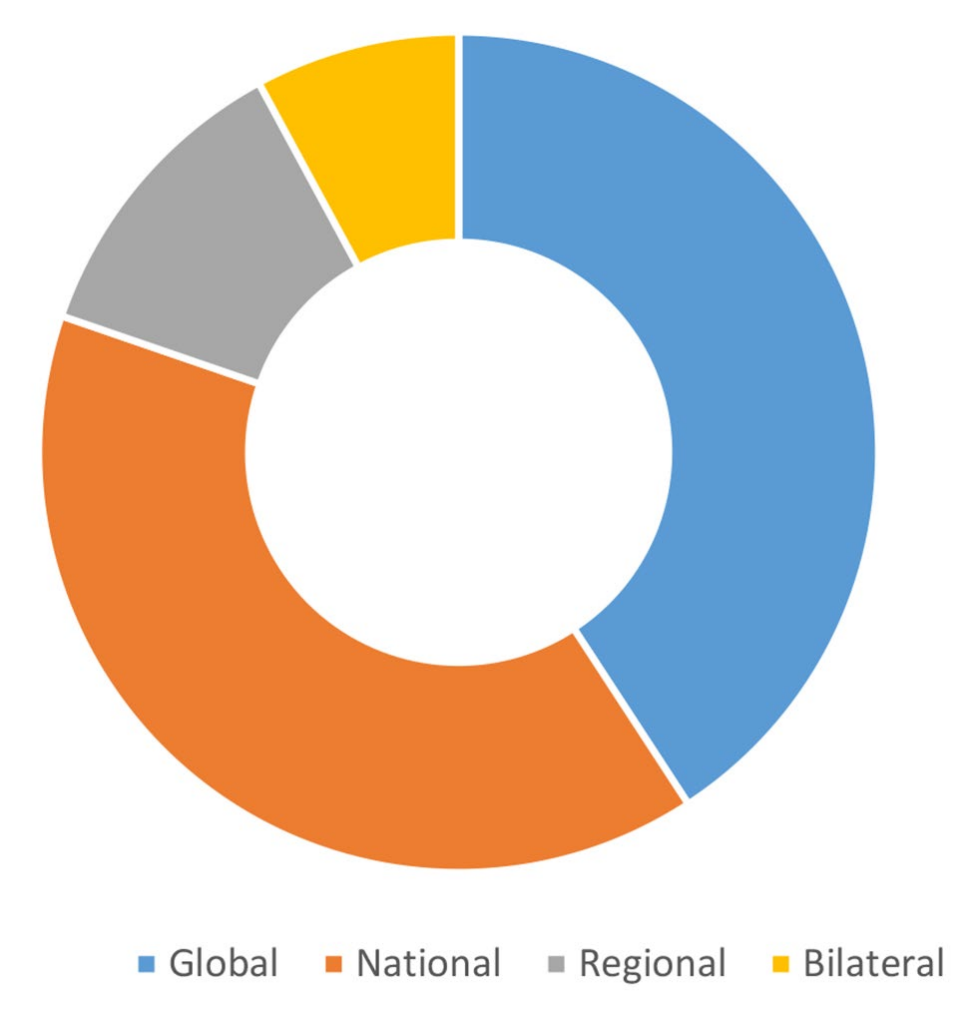
Level of policy

Studies examined different levels of trade policymaking, including interactions between levels. The majority of studies at the global level (See Fig. 3) examined multilateral trade policymaking at the WTO, including the WTO Doha Declaration on TRIPS and Public Health (n = 12), the WTO Paragraph 6 decision on TRIPS (n = 2) and the WTO TRIPS Council (n = 1). Other global studies included interactions between the WTO and WHO, Food and Agriculture Organization (FAO), World Food Program (WFP), the United Nations, and Codex Alimentarius Commission.

**Fig. 4:**
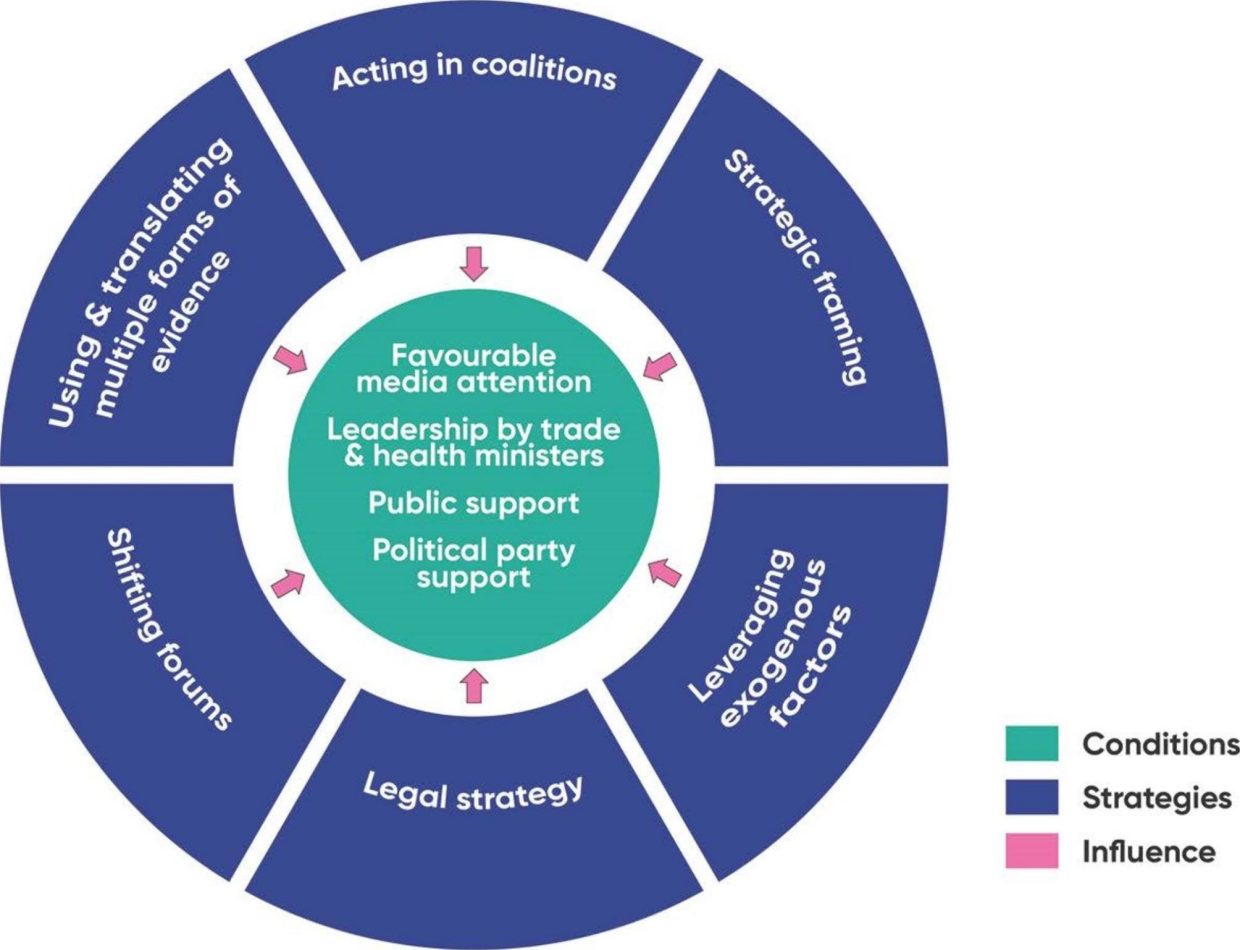
Strategies and conditions

Regional trade policymaking studied in the included papers included the European Union (EU (n = 4), Trans Pacific Partnership negotiations (n = 4), Anti-Counterfeiting Trade Agreement (ACTA) negotiations (n = 2),North American Free Trade Agreement (NAFTA), the Secretariat of the Common Market on Eastern and Southern Africa, Association of Southeast Asian Nations (ASEAN), and Regional Comprehensive Economic Partnership (RCEP) trade negotiations (all n = 1). All bilateral agreements studied involved the US. Studies focused on trade policymaking at the national level (including the development of national positions in specific trade negotiations and more general national trade policymaking) included studies of Australia (n = 6), Thailand (n = 5), Peru (n = 4), the USA (n = 1), Brazil (n = 1), Columbia (n = 1), one study of Fiji, Samoa and Tonga, and one study of Pakistan, Uganda and the Philippines.

Interactions between different levels of policymaking were also in focus in a smaller number of studies. These included interactions between the WTO TRIPS Council and regional ACTA negotiations [50], and intergovernmental organisations influencing national positions taken in bilateral trade agreements (e.g. the influence of the United Nations Committee on Economic, Social and Cultural Rights on Ecuador’s position in the US-Ecuador bilateral negotiations) [51]. Other studies examining interactions between the global and national included studies examining interactions between the WTO dispute settlement system and national policymaking, the WTO Technical Barriers to Trade (TBT) committee and national policymaking, and national policymaking shaping country positions taken at the WTO [52].

## Strategies and conditions enabling elevation of health goals in policymaking

**Fig. 5:**
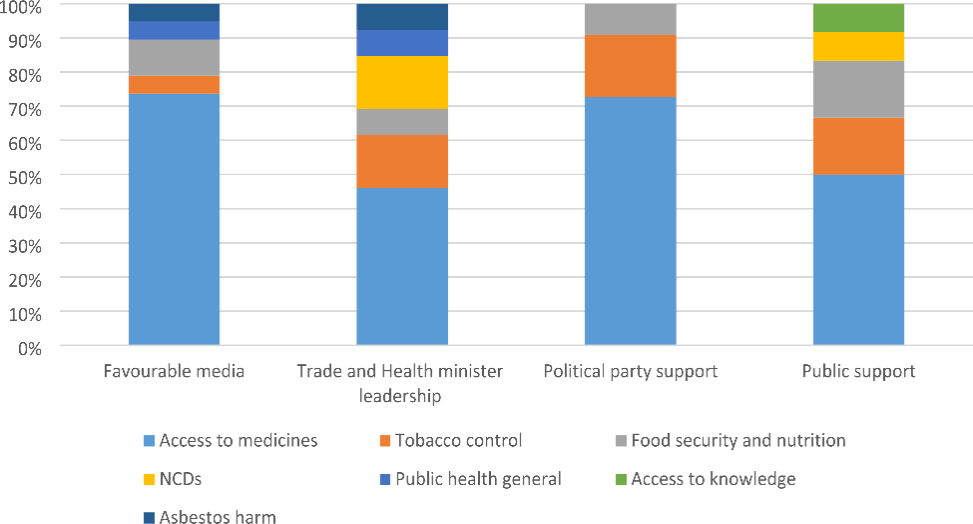
Conditions shaping elevation of health goals

Our thematic analysis identified four enabling conditions for the elevation of health in trade-related policymaking: favourable media attention, leadership by trade and health ministers, public support, and political party support. These were four common factors that were reported as influential on policymaking but were outside of the control of policy actors seeking to elevate health onto the agenda. These policy actors included civil society organisations, government officials from health ministries, academic experts and lawyers, and intergovernmental officials. Importantly, it was not essential for all conditions to be met for health to be elevated onto trade related policy agendas. Rather, these indicate that these conditions were often influential on policymaking. 62% of studies identified one condition, 22% two conditions, 16% three conditions, and no studies had all four conditions. The distribution of identified conditions by health issue in the studies is shown in Fig. 5. We identified six strategies successfully used by advocates to influence these conditions: using and translating multiple forms of evidence, acting in coalitions, strategic framing, leveraging exogenous factors, legal strategy, and shifting forums. Likewise, not all strategies appeared to be necessary. These conditions and strategies are represented in Fig. 4. We explain these conditions and strategies in the sections below.

### Favourable media attention

Favourable media attention was identified in 18 studies as a key condition for elevating health goals in trade-related policymaking [13, 37, 46, 52–65]. Favourable media attention was intimately connected to public opinion and political party support (see further sections below) [37, 46, 53, 55, 57–59]. For example, favourable international media attention was identified in three studies as a key condition in shaping the WTO Doha Declaration negotiations in favour of access to medicines [53, 57, 59]. A ‘sharp spike’ in international media reporting linking intellectual property (IP) to access to medicines was found to be a ‘substantive precipitant’ for the commencement of negotiations [59]. The ability of advocates to capture favourable mainstream media attention within high-income countries was also reported as important for increasing developing country gains in these global negotiations [53]. Front page reporting by the *New York Times* on generic firms’ significant price reductions for antiretroviral drugs was reported as key to ‘completely transforming’ the global debate on HIV and access to medicines surrounding the WTO negotiations [53].

Mainstream media reporting was also identified as important for causing reputational damage to actors who opposed public health goals in trade-related policy [37, 46, 55, 58]. Several studies reported on the dynamics shaping the pharmaceutical industry lawsuit against South Africa in the 2000s over its compulsory licensing legislation. Mainstream media uptake of pro-access to medicines framing caused reputational damage to the then US Clinton Administration, who supported the pharmaceutical industry litigation, eventually leading to the US withdrawing support for the industry [37, 59]. The pharmaceutical industry litigation itself became a ‘news peg’ for mainstream media reporting on patents, IP, and access to medicines [55], which was also found to have damaged the reputation of the pharmaceutical companies, leading the companies to eventually withdraw their dispute [46, 55, 58].

Favourable and frequent media attention was also identified as critical to influencing international public opinion in bilateral trade disputes. One study of Brazil, for example, found that favourable media attention in support of Brazil’s access to medicines was a key condition leading to the US withdrawing a formal complaint against Brazil at the WTO [60]. At the national level, media attention favourable to health was also found to have shaped public opposition to TRIPS-plus IP rules in a proposed regional trade agreement [54].

### Trade and health minister leadership

Leadership by trade and health ministers (or their equivalents) was also found to be crucial for elevating health goals in trade-related policymaking in eighteen studies [10, 23, 25, 31, 38–40, 52, 57, 60, 63, 64, 66–71]. Leadership by trade ministers in favour of health goals was identified in national settings [69], in trade negotiations [49] and at the WTO [67]. Leadership by health ministers was found to be key in several studies for strengthening trade negotiators’ positions in trade negotiations and in prioritising health in domestic trade-related policy [23, 31, 60, 63, 64, 68–71]. During the Trans-Pacific Partnership(TPP) negotiations, for example, Australia’s Health Minister was found to play a crucial leadership role in ‘trade-proofing’ Australia’s tobacco plain packaging policy in the face of tobacco industry disputes [69]. In contrast, four studies found barriers to the involvement of health officials in trade negotiations, which were constraining for elevating health onto the agenda [10, 13, 31, 72].

Intra-governmental collaboration between trade and health departments facilitated the inclusion of health goals in government trade mandates in eleven studies. Intragovernmental mechanisms for trade and health sector collaboration were found to be key for: Peru’s development of “red lines” on access to medicines and IP in FTA negotiations [66, 71]; Australia’s leadership on tobacco control in the face of trade disputes, which included shared drafting of Australia’s tobacco plain packaging law [10, 68, 69]; Canada’s trade ban on asbestos [68]; Uruguay’s defence of tobacco legislation [70]; Brazil’s trade ban on tobacco flavourings and additives [68]; Thailand’s health impact assessments of proposed trade negotiations [38]; and LMICs’ use of TRIPS flexibilities to support access to medicines objectives [57]. In Ghana, ongoing collaboration between trade and health ministers led to a joint food standards policy to limit the amount of fat in meat in response to rising imports of low-quality fatty meat cuts [40]. Similarly, studies of trade bans and trade-proofing health policy in Fiji and Samoa identified collaboration between the Ministry of Health and other ministries as crucial [25, 39].

### Public support

Public support (i.e. domestic salience of the health issue) was identified in eleven studies as a supportive condition, particularly for domestic trade policy and national positions taken in trade negotiations [23, 36, 39, 40, 49, 58, 60, 63, 70, 73, 74]. Public opposition to increased IP enforcement in the EU prevented the signing of ACTA [73]. Interestingly, it was found that high public salience incentivised more actors to campaign against ACTA, which further increased its public salience, generating an “attention cascade” effect [73]. Negative public opinion was found to contribute to the US withdrawing its trade dispute against Brazil regarding Brazil’s IP law [60]. Indeed, Brazilian negotiators reported that their strategy to defeat the US was focused on influencing US domestic public opinion through a multi-pronged strategy (cited in p. 136 [63]). Similarly, public support for access to medicines was reported as key to shifting the EU, Dutch, German, and French governments to oppose pharmaceutical industry litigation against South Africa [58]. At the national level, public opposition to the dumping of fatty meats by high-income countries in the Pacific was a key condition for the development of a health-related trade ban in Fiji [39]. Similarly, public concern over rising imports of low-quality fatty meats was an important factor shaping the development of a food standards policy in Ghana [40].

### Political party support

Political party support for health was identified as an important condition in ten studies [23, 37, 39, 46, 57, 66, 70, 71, 75, 76]. In the USA, two studies reported on how NGO campaigning in the 2000s led to access to medicines becoming a wedge issue in federal elections, leading to commitments from major political parties [37, 46]. Two other studies found that when the Democrat party gained political power in the US Senate in the mid-2000s, a ‘New Trade Policy for America’ was issued, which led to the amendment of IP proposals in negotiations with Peru and Colombia to remove several TRIPS-plus provisions [66, 71]. Likewise, the opposition Australian Labor Party in Australia insisted on an amendment to the implementing legislation of the Australia-United States Free Trade Agreement (AUSFTA) intended to prevent spurious patent claims that could delay the market entry of generic medicines [76]. Newly elected parliamentary majorities of Indigenous peoples in Ecuador and Bolivia walked away from the US-Andean Free Trade Agreement negotiations in defence of access to medicines [57]. In Fiji, a Labour government elected in mid-1999 was found to be much more willing than the previous government to intervene in the market to improve health [39].

### Strategies to influence the conditions

**Fig. 6:**
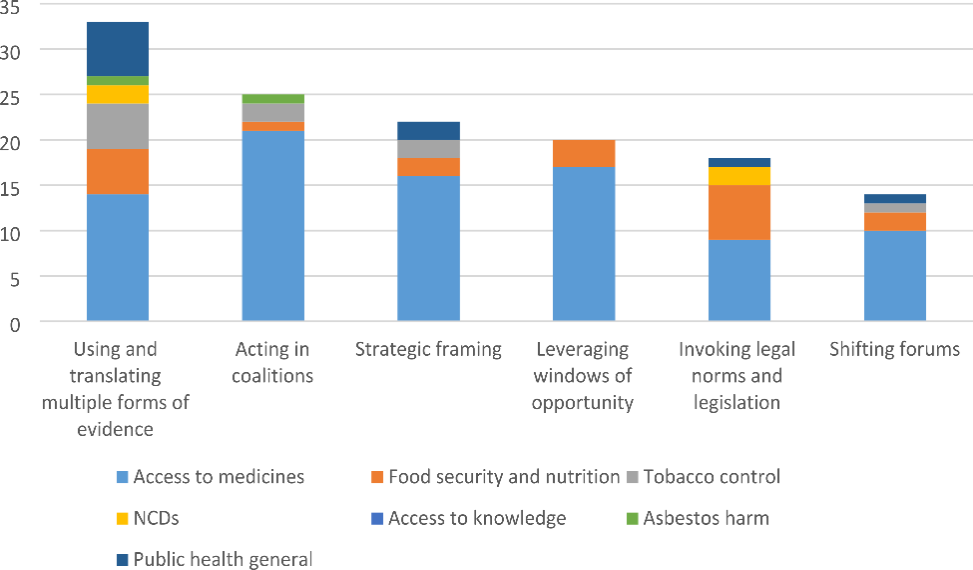
Strategies shaping elevation of health goals

We identified six strategies used by policy actors to influence these conditions in order to elevate health goals in trade-related policymaking: *using and translating multiple forms of evidence; acting in coalitions; strategic framing; leveraging exogenous factors; invoking legal norms and legislation; and shifting forums*. Again, not all strategies appeared necessary in every case. Twenty-nine studies identified one strategy, nineteen studies identified two strategies, nine studies identified three strategies, six studies identified four strategies, two studies identified five strategies, and no studies identified all six strategies. The distribution of strategies by health issue is shown in Fig. 6.

#### Using and translating multiple forms of evidence

The most common strategy, identified in 27 studies, was using and translating multiple forms of evidence, including health, economic, and public opinion data, to support health goals in trade-related policymaking [10, 12, 23, 25, 37, 49, 54, 56, 58, 61, 64, 66–68, 71–73, 77–86]. Of the different types of trade policymaking studied, using and translating evidence appeared to be particularly identified in trade disputes, trade challenges, and trade negotiations. This is likely due to the evidentiary nature of trade disputes at the WTO, where member states must defend their measures using legal argument and evidence.

The use of health impact assessments (HIA) as technical evidence was found to have influenced attention to access to medicines in national positions taken in trade negotiations in Australia [54], Thailand [61, 66], and Peru and Colombia [71]. A Peruvian HIA that raised concerns for TRIPS-plus IP in the Peru-US FTA negotiations was found to persuade the US to remove some of these measures following a change in government [66]. Public health experts’ debunking of industry claims was also highlighted as a ‘pivotal turning point’ for advocacy that led to Canada’s trade ban on asbestos [64]. In addition, two studies highlighted NGO evidentiary challenges to pharmaceutical industry claims regarding links between IP and research and development expenditure as influential in defending South Africa’s medicines law against a US trade challenge [58, 84]. The UN General Secretary’s High-Level Panel on Access to Medicines (2015), which condemned threats to undermine the use of WTO TRIPS flexibilities, was also reported as influential in reasserting access to medicines as a right to health at the WTO [77]. Furthermore, one quantitative study found that the higher the domestic health concerns of a country, the weaker the effect of that country’s commitments to IP protection [78]. Two studies reported on the use of personal stories regarding health and access to medicines as informing supportive media attention and public opinion [23, 49]. Four studies reported on health evidence provided by WHO as important for trade-proofing tobacco plain packaging policy [68–70, 85]. Amicus briefs (i.e. written legal submissions) prepared by the WHO Framework Convention on Tobacco Control (FCTC) Secretariat, for example, were found to be an important source of evidence in support of Uruguay’s defence of its tobacco control measures at the WTO [79].

Seven studies found that economic evidence (i.e. the costs and economic benefits) was used to elevate health goals – particularly in trade disputes regarding tobacco [67, 68] and in supporting prioritisation of access to medicines in trade negotiations [10, 23, 37, 71, 80]. One study highlighted the generation of public opinion data commissioned by NGOs as influencing EU members of parliament not to support stringent IP measures in the ACTA agreement [73]. In contrast, a lack of strong evidence was found to undermine some public health claims in trade-related policymaking [10, 12, 86].

Finally, the importance of translating evidence for a wider public audience was highlighted explicitly in two studies of trade negotiations where LMICs, such as Thailand and Brazil, successfully defended access to medicines concerns [80–82]. Likewise, one study reported that the lack of translation of evidence was a barrier to health goals [56].

#### Acting in coalitions

Twenty-five studies identified acting in coalitions as a key strategy to advancing the prioritisation of health in trade-related policymaking [23, 36–38, 46, 49, 50, 53, 56–59, 64, 68, 70, 71, 84, 87–94]. Coalitions included those between LMICs; between civil society organisations; and broader coalitions involving government officials, civil society and other supportive organisations and experts. LMIC coalitions were identified as particularly important in trade negotiations. Civil society coalitions were identified across all trade categories, often involving health NGOs working with other civil society actors, including trade unions, public interest organisations and sovereignty movements in LMIC, and were particularly prominent in studies of trade negotiations and domestic implementation.

LMICs acting as a bloc in trade negotiations was identified in five studies as important to the outcome of the Doha Declaration [46, 53, 59, 87, 89] and the subsequent Decision on Paragraph 6 [94]. By acting as a bloc, LMIC could contest the power of well-resourced high-income countries. For example, LMICs acting as a bloc voicing opposition to the ACTA negotiations (which they were not party to) at the WTO TRIPS Council was found to be influential on high-income countries abandoning the agreement [50]. Likewise, LMICs acting in a negotiating bloc during the RCEP negotiations was highlighted as important for elevating access to medicines concerns as high-income countries were outnumbered [23, 49].

Civil society coalitions [37, 88] and coalitions between civil society actors and LMICs [36, 53] were identified as significant in studies of global, regional, and bilateral trade negotiations. Even though these actors were not parties to the negotiations (which are state based), they were able to work across contexts to strategize, frame and influence media and public opinion, putting pressure on countries to pay attention to health concerns. The multiple actors in various coalitions are demonstrated by Drahos’ analysis of the WTO Doha Declaration [36]:

“An Africa Group that joined with a large coalition of developing countries that included Brazil and India, that drew on the power of Northern NGOs to work the Northern mass media, that gained the quiet support of some European states, that drew on independent technical expertise to evaluate draft text, that gained resources from Geneva-based NGOs was a group strengthened by many ties…. If TRIPS was about a form of networked governance in which the powerful built ever larger circles of consensus in the shadow of credible threats of trade coercion, the Doha Declaration was about the weak networking networks that surrounded and eventually isolated the US and in the final instance its pharmaceutical industry.” [36].

Three studies found that NGO coalitions were influential in shifting the EU’s position on IP and access to medicines [56, 58, 84]. Three studies found civil society coalitions in Thailand, South Africa, Peru, and Colombia were influential for drawing attention to access to medicines concerns in trade negotiations and the implementation of trade rules [57, 71, 90]. Likewise, the formation of a regional civil society network monitoring the RCEP negotiations was found to be a key factor in raising concerns around access to medicines and generating opposition to several TRIPS-Plus measures [49].

Informal coalitions between government health officials and civil society actors were identified in four studies [38, 68, 70, 91], and informal coalitions between civil society actors and generic pharmaceutical firms were identified in five studies of the Doha Declaration [37, 53, 91–93]. One study reported on the Pan American Health Organization (PAHO) as a key coordinator of an informal coalition of domestic Health Ministries in Ecuador, Peru, and Columbia that successfully elevated access to medicine concerns on the agenda in the context of the ANDEAN FTA negotiations [52, 71]. Finally, a transnational coalition of asbestos victims, health NGOs, trade unions, and health experts was identified as crucial for securing Quebec’s trade ban on asbestos on health grounds [64].

#### Strategic framing

Twenty-one studies identified supportive framing as enabling the elevation of health goals in trade-related policymaking [12, 23, 36, 37, 46, 49, 51, 53, 55, 57, 59, 60, 62, 63, 65, 68, 69, 73, 74, 84, 90]. Policy actors use frames often strategically, to focus attention to a particular issue and persuade others of its importance [12]. Framing was an important condition across all types of trade policymaking, particularly in trade negotiations and ‘trade proofing’ health policy. Policy actors developed and invoked different frames to consolidate campaigns, mobilise media attention, and generate public support for health issues. NGOs were prominent actors in using framing to elevate health on the trade policy agenda.

Access to medicines was identified as a powerful framing and norm, in its own right, in eleven studies [23, 36, 37, 49, 53, 55, 57, 59, 60, 74, 90]. This framing shifted debates on IP and trade from private goods to public health and enabled NGOs and low-income countries with comparatively less material power to exert discursive power over trade agendas [36, 49, 59, 60]. Brazil’s government, for example, used access to medicines framing to shift public opinion internationally, ultimately leading to the US withdrawing its formal dispute against Brazil regarding its IP policy [63].

Part of the success of this framing was its simplicity – providing a simple story about access to medicines and IP which ‘no individual, country or organization could be seen to be [against]’ [36, 51]. Reducing complexity in the WTO trade law down to a simple statement that “nothing in the TRIPS agreement shall prevent public health” was identified in several studies as an important framing that enabled coalitions, media attention, and public support [36, 46, 59, 65, 87]. In contrast, a lack of simple framing was reported as a barrier to health goals in some studies [10, 72].

Human rights and right to health framing were also successfully used, particularly in national cases [58, 84]. In South Africa, for example, NGOs’ use of human rights framing in affidavits and wider human rights campaigning, was found to have shaped public support and media uptake of pro-health framing [84]. In Australia, tobacco control advocates successfully framed tobacco as a unique health harm to convince policymakers to support tobacco plain packaging in the face of tobacco industry challenges [69]. Economic framing was found to be useful in some studies. In a case of food security and trade, for example, policy actors framed food pricing as a balance of payments issue, aligning with the dominant economic framing in order to bring food insecurity onto the General Agreement on Tariffs and Trade (GATT) agenda [62]. Economic framing was also aligned with the language of market access for generic medicines, enabling non-health actors to support reduced-IP commitments based on economic arguments rather than health arguments [23]. Economic framing was found to be constraining, however, for consideration of the wider social determinants of health [12].

#### Leveraging exogenous factors

Twenty-one studies identified leveraging exogenous factors outside trade policy processes as a factor that influenced media and public opinion, particularly in trade negotiations [37, 39, 46, 47, 49, 53, 55, 58, 62, 63, 65, 66, 71, 80, 87, 89, 92, 93, 95–97]. Exogenous factors included the rise of HIV as a health and security crisis to draw attention to the issue of access to medicines [87, 92, 93, 95], concerns over product dumping to advance prioritisation of nutrition in domestic trade-related policymaking [39], and concerns over food pricing and global harvest levels to advance food security on the agenda at the WTO [62].

Eight studies documented the anthrax scare in the USA and the SARS epidemic during the time of the WTO Doha negotiations as events seized upon by several actors to draw media attention to the issue of access to medicines and IP [37, 46, 47, 53, 63, 65, 87, 89]. Four studies identified domestic elections as events leveraged by NGOs to secure political party support [37, 46, 58, 71]. The seizure of generic medicines in transit in Europe in 2008 was invoked by LMICs at the WTO TRIPS Council in their opposition to the regional ACTA agreement [96]. The suspension of controversial IP measures in the then TPP negotiations following the US withdrawal in 2018 was found to be an important exogenous event that supported access to medicines concerns in the RCEP negotiations [49].

#### Legal strategy

A fifth strategy, identified in 18 studies, was using different forms of legal strategy to support elevation of health goals [13, 23, 32, 40, 49, 56–58, 60, 65, 66, 68, 69, 79, 81, 86, 98, 99]. The three forms of legal strategy identified in the studies were: the use of non-trade treaties in trade law, embedding the legal right to health in domestic law, and using law in developing policy. Legal strategy appeared to be particularly important for trade disputes and challenges, trade bans, and trade-proofing health policy.

Six studies identified the use of the FCTC (a non-trade treaty) to defend tobacco control measures, including Australia’s defence of tobacco control measures in trade disputes [23, 32, 68, 69], Brazil and Canada’s defence of their tobacco control bans on tobacco additives and flavourings [68], and Uruguay’s defence of its tobacco control measures [70, 79]. WTO panels have cited the FCTC as an authoritative source of evidentiary support for the impact of tobacco control measures on population health [68, 79]. Similarly, five studies found the Doha Declaration to have enabled LMICs to assert their right to use flexibilities in implementing TRIPS in ways that protect access to medicines [23, 49, 57, 60, 86]. In contrast, analysis of front of pack nutrition labelling by Thow et al. [98] found that the absence of a Codex standard prioritising health meant that national governments were ‘likely to be vulnerable to challenges at the WTO’.

Three studies identified a domestic legal right to health as influencing trade policymaking. Thailand’s Constitution, for example, mandates transparency in the process of trade negotiations and embeds human rights principles that grant legal power to examine trade agreements for potential violations of human rights [38, 66]. Similarly, South Africa’s constitutional framework entrenches the right to health, and was identified as an important legal source for health actors to assert the right to access to medicines [58].

Legal strategy included filing pre-grant oppositions against pharmaceutical patent applications [81], and obtaining support from legal scholars domestically [69] and in global negotiations [65]. Using trade law principles such as non-discriminatory approaches was highlighted as key to the successful development of an innovative food standards policy in Ghana [40]. Embedding policies within a larger comprehensive suite of public health responses also meant that opponents were less able to invoke WTO rules referring to ‘less trade restrictive alternatives’ to oppose governments’ public health policies [99]. The primacy of law could be a double-edged sword, however. Analysis of EU trade policymaking on IP and access to medicines found that arguments that were inconsistent with the legal and epistemic foundations of the international trade regime were ‘inconceivable’ to trade officials and effectively sidelined [56].

#### Shifting forums

The final strategy, identified in 14 studies, was shifting forums - where advocates for health manoeuvred across different institutional forums to put pressure on trade-related policy domains [37, 46, 47, 51–53, 59–63, 66, 70]. This strategy has been documented in studies of corporate actors in trade policy [100], and was identified in our sets of studies of national level policymaking, including national positions taken in trade negotiations, trade negotiations between countries, and domestic implementation of trade-related policy.

At the national level, the Thai NGO FTA Watch shifted the focus of its advocacy from the trade policy domain to the National Human Rights Commission of Thailand, an independent quasi-government institution, to call for a human rights impact assessment (HRIA) of Thailand’s proposed trade agreement with the USA [61, 66]. The Commission conducted the HRIA in 2006, recommending that “intellectual property protection relating to drugs and public health services should not be considered in the bilateral trade negotiations” [61]. The report appeared to influence subsequent Thai law with HRIA becoming entrenched in national policymaking [61, 66].

Several studies documented forum shifting at the global level. Three studies highlighted the importance of Zimbabwe shifting the issue of access to medicines onto the WTO trade agenda through the appointment of Zimbabwe’s Ambassador Boniface Chidyausiku to the WTO TRIPS Chair in the early 2000s (which informed the negotiations for the Doha Declaration) [46, 53, 59]. Forum shifting food security issues to the UN led to the adoption of the UN Special Rapporteur’s proposal for waiving agricultural subsidy rules for public food stockpiling at the WTO [62]. Similarly, the FAO was found to be instrumental in mobilising LMICs to put food security on the GATT negotiating agenda, which led to decisions on safeguarding food security [62]. The WFP has also actively influenced the WTO on food aid rules – launching media campaigns during the mid-2000s in support of LMICs being able to draw on surplus food commodities for food security [62].

Forum shifting from the WTO to the WHO and its World Health Assembly was also identified as an important strategy leading up to the Doha Declaration [37]. Two studies reported on attempts by NGOs and LMICs to shift the issue of access to medicines and IP to the WHO through the Revised Drug Strategy (1998) [37, 47]. The WHO forum was more favourable to health arguments, and the final strategy called on member states to ensure equitable access to essential drugs and to review options under trade agreements to safeguard these medicines. NGOs then used this normative frame to call on WTO member states to support public health flexibilities in TRIPS [37, 47].

Forum shifting between institutions at the national and global level was identified in five studies. One study, for example, reported on how the global NGO 3D shifted to the United Nations Committee on Economic, Social and Cultural Rights (UNCESR) amidst to prosecute its concerns for access to medicines regarding the proposed Ecuador-US FTA. In considering the complaint, the UNCESR recommended that Ecuador consider the right to health and flexibilities for ensuring access to medicines and the right to health for all. 3D circulated these recommendations to human rights, development and access to medicines networks in the Andean region and the recommendations were used by Ecuadorian domestic civil society organisations. The Ecuadorian Trade Minister responded by dropping a draft decree that contained TRIPS-plus rules, and Ecuador did not agree to TRIPS-plus rules in the agreement [51]. Similarly, when Brazil faced a formal US trade dispute over its antiretroviral policy, it forum shifted to the WHO, focusing on resolutions promoting access to medicines and HIV/AIDS. As former Brazilian Health Ministry Diplomat Marcos Viana noted:

“The idea of moving our resolutions through the UN agencies was important for shaping global public opinion in our favour. So we developed a strategy at the World Health Assembly to introduce medicines resolutions. At the Commission on Human Rights, we pushed through that resolution that documented that access to medicines was a fundamental human right” (cited in [63]).

In response to Brazil’s diplomacy, the UN High Commissioner on Human Rights issued a report highlighting the Brazilian case as a dilemma for countries promoting access to medicines. Not long after, the United Nations General Assembly (UNGA) held a Special Session on HIV/AIDS at which the United States Trade Representative (USTR) formally dropped its trade dispute against Brazil [60, 63].

In a similar forum shifting move, as part of its defence of tobacco plain packaging against tobacco firm challenges, Uruguay used the WHO FCTC Conference of Parties to table the Punta Del Este Declaration, declaring the rights of sovereign countries to prioritise public health regulations over trade agreements [70].

Related to the focus on forums was the finding from one quantitative study of US bilateral FTAs, which found that the longer trade agreement negotiations go on for, the fewer commitments there are on IP related to pharmaceuticals [97]. This suggests that strategies that delay negotiations mean fewer commitments which can negatively affect public health.

## Discussion

For more than a decade, scholars and advocates have decried the fact that health is given low priority in trade policy[31, 33, 101]. To date, there has not been a comprehensive review of the strategies and conditions that have enabled the elevation of health in trade policy across different contexts. Our systematic search of the literature identified 65 studies spanning several different types of trade-related policy making, which together represent a treasure trove of lessons for policy actors and health advocates. A majority of studies (59%) focused on access to medicines, with smaller numbers of studies addressing food supply and nutrition, tobacco control, non-communicable diseases, access to knowledge, asbestos harm and public health in general. This finding is unsurprising given the high-profile nature of battles over IP and access to medicines globally. On the one hand, this may suggest that access to medicines is more successful in entering the trade agenda than other public health issues. Yet, access to medicines remains at risk, with many governments continuing to sign bilateral and regional trade agreements that include TRIPS-plus measures [102]. The recent failure to secure a substantive waiver on intellectual property for COVID-19 pandemic vaccines and products at the WTO is a case in point [11].

A more nuanced interpretation is that access to medicines has become a health and trade norm in some contexts. Our analysis shows that, since the WTO Doha Declaration on TRIPS and Public Health, access to medicines has become an established frame in many high, low, and middle-income countries and in global debates, which has influenced media reporting and public opinion. Furthermore, economic framing on the costs of medicines has been used in a supportive way in different contexts [10, 23, 37, 71, 80], benefiting from the dominant neoliberal paradigm. The studies demonstrate that evidence remains important, but must be translated so it is readily understood by policy actors. The studies also demonstrate the importance of coalitions, and the role of patient access groups and treatment access groups in particular, leading globally networked movements across countries, agreements, and contexts [23, 36–38, 46, 49, 50, 53, 56–59, 64, 68, 70, 71, 84, 87–94]. The temporal dimension to these studies on access to medicines, over more than 20 years, demonstrates that once one or more political conditions are established, such as a supportive political party, they are then leveraged in subsequent trade debates, keeping access to medicines on the agenda [23]. However, political dynamics mean governments, leaders and priorities change, forcing advocates to continually draw on their networks and framing strategies to maintain pressure.

For other health issues, it is noteworthy that trade and health ministers’ (or equivalent) leadership and public support appeared as influential conditions (i.e. without the need for favourable media attention or strong political party support) [10, 25, 38–40, 68–70]. Using legal strategies and using different forms of evidence also appeared to be the two strategies influential for issues other than access to medicines (and in particular tobacco and food and nutrition) to rise on the trade policy agenda [40, 64, 67–70, 79, 85, 99]. This has likely been shaped by the wider historical context of tobacco industry lawsuits and trade challenges regarding food and nutrition policy at the WTO, as well as an absence of more prominent frames and global networks for these public health issues (see more below on strategies).

Included studies were unevenly spread across levels of analysis, with the majority focusing on negotiations at the WTO and national level trade policymaking, and smaller numbers examining regional or bilateral agreements. This suggests that it may be more difficult to get health onto the agenda for regional or bilateral agreement negotiations occurring outside the WTO umbrella. Unlike the WTO, which publishes agendas and draft resolution text and has NGO and other actors formally monitoring some aspects of the negotiations (although this is not the case for infamous Green room discussions), bilateral and/or regional agreements are largely negotiated in secret. Furthermore, for many countries, elected representatives cannot see treaty text until agreements are signed. It is also difficult to study these agreements. If countries decide to not include a provision in a trade agreement because it may negatively affect public health, it may be that public health is on their agenda but this may not be evidence to the outsider. Indeed, many of the studies included in our review suggest that advocacy often results in small wins, for example, preventing or mitigating measures from being included in an agreement in defence of public health goals [49].

We found there were four common conditions that enabled prioritisation of health across the studies: favourable media attention, leadership by trade and health ministers, public support and political party support. These findings highlight the importance of politics. Generating and using evidence is an important strategy (see below), but favourable media attention, public support, political party support, and ministerial support, are all political conditions that require different strategies. Favourable media attention, for example, was not explicit in much of the existing trade and health governance literature. This review indicates it is an important political condition, particularly for materially weak actors to influence trade agendas. Furthermore, it is important to note that not all conditions appeared necessary to influence trade agendas. Not one single study contained all conditions. Furthermore, the conditions were intimately connected – for example, favourable media attention was found to influence political support and public opinion [37, 46, 53, 55, 57–59]. We were not able to capture the depth of the complexity of these relationships by reviewing these 65 studies, rather we present a suite of conditions and strategies that were commonly identified as influential.

For those health issues not including access to medicines, Trade and Health minister leadership and public support appeared necessary [10, 25, 38–40, 68–70]. This is likely due to a lack of strong frames and established coalitions, and suggests that these conditions may be key targets for resource-constrained policy advocacy. Favourable media attention and political party support were more evident within the studies on access to medicines, which provides lessons for a multi-pronged approach to generating other health issues onto trade agendas.

Our findings suggest that six strategies have been successfully used by policy actors to enable these conditions: using and translating multiple forms of evidence, acting in coalitions, strategic framing, leveraging exogenous factors, legal strategy, and shifting forums. Like the conditions, not all strategies were needed for success. Rather, they were informed by the particular context. For example, legal strategy and using different forms of evidence appeared to be important strategies for tobacco and food and nutrition [40, 64, 67–70, 79, 85, 99]. This is likely shaped by the context of tobacco disputes and trade challenges at the WTO, leading to a legal defence of health. It is also important to note the context for the use of evidence. For example, while we found that HIA have been used successfully to advance health, others exploring impact assessments more broadly and their use in the EU have found that they can be used to advance corporate interests depending on how they are designed [103].

One surprising finding was that our analysis revealed that the same strategies and conditions broadly applied across the different types of trade-related policymaking. Strategic framing, as a strategy, was consistent across all the trade-related categories, as was acting in coalitions. There were also, however, some key differences in the prominence of different strategies and conditions in different types of trade-related policymaking. LMIC coalitions were particularly important for trade negotiations, while legal strategy and using multiple forms of evidence were particularly important for trade disputes and trade challenges. Health and Trade Minister support were particularly strong for national policymaking including trade bans and trade-proofing health policy.

These strategies add to the existing literature on the importance of framing and coalitions in trade and health literature [10, 12, 16, 17, 19, 20, 22, 31, 72]. They also point to the need for political strategies, suggesting capacity building for trade and health should include capacity building for political strategies on what frames, actors, and forums can assist health and policy advocates to be heard. Indeed, these strategies appear as influential for policy actors to elevate health onto the agenda in less than ideal governance contexts, acknowledging ongoing challenges for greater transparency, participation, and accountability [21, 30]. The findings could be applied to interrogate other areas of the commercial determinants of health, where health goals are on the periphery of economic policymaking.

### Limitations

This study is limited to peer reviewed studies published in English and does not capture studies published in other languages due to the authors’ native language constraints. The screened studies were captured from the search terms, and it is possible that studies not using these terms in the abstract, keywords or title were missed. As we were relying on how authors reported what was important in the included studies, there are limits on the extent we could probe relationships between conditions and between strategies, and it was not possible to capture the depth of individual nuance of each of the studies in the thematic analysis.

## Conclusion

Despite accumulating evidence of the health impacts of trade policy, health is often on the periphery of trade policy agendas. This review collated and analysed the literature on what has worked to enable the elevation of health in relation to trade across different forms of trade-related policymaking. Access to medicines is the dominant health issue in focus in these studies, likely reflecting the greater success for this health issue, although wins have still been small and ad hoc. We found four common conditions for elevating health: favourable media attention, leadership by trade and health ministers, public support and political party support. Six strategies were identified from the literature that shaped these conditions: using and translating multiple forms of evidence, acting in coalitions, strategic framing, leveraging exogenous factors, legal strategy, and shifting forums. The analysis demonstrates that while technical evidence is important, political strategy is necessary to influence the conditions for elevating health on trade agendas. The analysis provides lessons that can be applied to the wider commercial determinants of health where economic interests and health interests often collide.

### Electronic supplementary material

Below is the link to the electronic supplementary material.

Supplementary Material 1

## Data Availability

The datasets used and/or analysed during the current study are available from the corresponding author on reasonable request.

## Footnotes

1 Access to knowledge is an important social determinant of health, which can be affected by copyright and intellectual property rules included in trade policy (11).

## Abbreviations

ACTA: Anti-Counterfeiting Trade Agreement
ASEAN: Association of South-East Asian Nations
AUSFTA: Australia-United States Free Trade Agreement
EU: European Union
FAO: Food and Agriculture Organization
FCTC: Framework Convention on Tobacco Control
HRIA: Human Rights Impact Assessment
IP: Intellectual property
LMIC: Low and middle-income countries
NAFTA: North American Free Trade Agreement
NCD: Noncommunicable disease
NGO: Nong-government organisation
RCEP: Regional Comprehensive Economic Partnership Agreement
TRIPS: Trade-Related Aspects on Intellectual Property Rights
TBT: Technical Barriers to Trade Committee
TPP: Trans Pacific Partnership
UN: United Nations
UNESCR: United Nations Committee on Economic, Social and Cultural Rights
UNGA: United Nations General Assembly
USTR: United States Trade Representative
WFP: World Food Programme
WHO: World Health Organization
WTO: World Trade Organization
