# Supplementary material for: Public health advocacy strategies to influence policy agendas: lessons from a narrative review of success in trade policy
Source: Global Health. 2023 Aug 23;19:60. doi: 10.1186/s12992-023-00960-7 (PMC10463651; doi:10.1186/s12992-023-00960-7)
Supplement: Supplementary file 1 — Supplementary Material 1 [file 12992_2023_960_MOESM1_ESM.docx]

| **Authors** | **Pub. Year** | **Focus and Level of analysis** | **Country(ies) studied  (or substantive focus if did not study specific country(ies)** | **Public health issue** | **Trade category** |
| --- | --- | --- | --- | --- | --- |
| Abbott, F.M. | 2002 | USA position, negotiation of Doha Declaration. | USA, negotiation of Doha Declaration | Access to medicines | Negotiation - global, regional, bilateral |
| Abbott, F.M. | 2005 | Developing countries' positions regarding the WTO General Council decision adopted on the Implementation of Paragraph 6 of the Doha Declaration on the TRIPS Agreement. | Global - WTO members | Access to medicines | National positions taken in negotiations |
| Abdel-Latif, A. | 2014 | Developing countries use of the WTO TRIPS Council as a forum to contest ACTA. | Developing countries (China, India and Brazil highlighted) in a group versus ACTA parties | Access to medicines | Negotiation - global, regional, bilateral |
| Baert, T. | 2016 | How the EU has sought to orient its trade policy to support its global health agenda. | The EU | Access to medicines | Negotiation - global, regional, bilateral |
| Baker, P.; Friel, S.; Gleeson, D.; Thow, A. M.; Labonte, R. | 2019 | How actors in the domestic Australian trade policy-making sub-system frame nutrition and implications for coherence between trade and nutrition policy in Australia. | Australia | Food - nutrition | National level policymaking |
| Baker, P.; Kay, A.; Walls, H. | 2015 | Key challenges to governing the health-trade nexus and advancing policy coherence in Asia. | ASEAN+3 countries: Cambodia, Indonesia, Laos, Malaysia, Myanmar, the Philippines, Singapore, Thailand, Vietnam, China, Japan, South Korea, and India | NCDs | Negotiation - global, regional, bilateral |
| Basheer, S. | 2018 | India's response to the WTO TRIPS Agreement through Section 3(d) of its patent law. | India | Access to medicines | Domestic implementation |
| Battams, S.; Townsend, B. | 2019 | Views of policy actors at the national and international level on challenges to achieving policy coherence between trade and health. | Australia, Malaysia, and Switzerland | NCDs | Negotiation - global, regional, bilateral |
| Bloche, M. G. | 2002 | The WTO's treatment of national health policies in disputes over members' treaty obligations for GATT Art, the SPS Agreement, and the TRIPS Agreement. | WTO system focus | Public health general, food -supply, access to medicines | Disputes and trade challenges |
| Blouin, C. | 2007 | Conditions in the national policymaking context important for advancing policy coherence between international trade policies and national health objectives. | Costa Rica, Malaysia, Argentina, Dominican Republic, Mexico, Thailand, the US, Sri Lanka, Secretariat of the Common Market on Eastern and Southern Africa (regional bloc), and Peru | Public health general | National level policymaking |
| Crosbie, E.; Sosa, P.; Glantz, S. A. | 2018 | The role of civil society and the transnational tobacco control network in Uruguay's successful defence of domestic tobacco packaging and labelling regulations against ISDS challenges from Phillip Morris International. | Uruguay | Tobacco control | Trade proofing health policy' - governance conditions |
| Crosbie, E.; Thomson, G.; Freeman, B.; Bialous, S. | 2018 | The role of legal preparation and support and a whole of government approach in the enactment and successful defence of Australia's domestic standardised packaging regulation against ISDS challenges from tobacco companies. | Australia | Tobacco control | Trade proofing health policy' - governance conditions |
| Crump, L.; Druckman, D. | 2012 | The triggers and consequences of parties' changes in position in two cases of multilateral intellectual property rights negotiations: the GATT Uruguay Round and the WTO Doha Ministerial. | WTO negotiations | Access to medicines | Negotiation - global, regional, bilateral |
| da Fonseca, E. M.; Bastos, F. I. | 2016 | Brazil's domestic implementation of IP protections required under the WTO TRIPS Agreement. | Brazil | Access to medicines | Domestic implementation |
| Deere, C. | 2009 | TRIPS implementation battles – globally, regionally and nationally. | National variation of TRIPS implementation and global negotiations. | Access to medicines | Domestic implementation |
| Drahos, P. | 2007 | Key lessons for developing countries in trade negotiations, derived from access to medicines issues in multi- and bi-lateral trade contexts (WTO, TRIPS, the Doha Declaration, and FTAs). | Developing countries | Access to medicines | Negotiation - global, regional, bilateral |
| Drope, J.; Lencucha, R. | 2013 | Strategies for progressing domestic tobacco control policies in the international trade agreement environment, based on learnings from disputes raised at the WTO's TBT committee in relation to domestic tobacco control policies in Canada, Brazil, the USA, and Australia. | Canada, Brazil, the USA, and Australia | Tobacco control | Disputes and trade challenges |
| Drope, J.; Lencucha, R. | 2014 | Norms-related tensions and developments between tobacco control and open trade in the GATT-WTO system, focusing on trade challenges to domestic tobacco control legislation in Thailand, Canada, the USA, Australia, and Brazil. | Thailand, Canada, the USA, Australia, and Brazil | Tobacco control | Disputes and trade challenges |
| Dür, A.; Mateo, G. | 2014 | Factors which contributed to civil society's successful campaign to stop the EU from ratifying ACTA. | The EU | Access to knowledge | Negotiation - global, regional, bilateral |
| Escobar-Andrae, B. | 2011 | The extent of IP protections beyond WTO-TRIPS requirements (i.e. 'plus' provisions) in bilateral trade agreements negotiated by the US during the 2000s, and the negotiation conditions associated with these commitments. Examined US agreements with 17 trade partner countries: Jordan, Singapore, Chile, El Salvador, Guatemala, Honduras, Nicaragua, Costa Rica, Morocco, Australia, Dominican Republic, Bahrain, Ecuador, Peru, Colombia, Oman, Panama, and Korea. | The USA | Access to medicines | Trade negotiations between two or more countries |
| Fairman, D.; Chigas, D.; McClintock, E.; Drager, N. | 2012 | Bilateral disputes between the USA and Brazil in relation to Brazil’s domestic stance on IP protection and HIV medicines, and the strategies and tactics deployed by Brazil that contributed to the US withdrawing its formal complaint to the WTO. | Brazil and the USA | Access to medicines | Disputes and trade challenges |
| Fairman, D.; Chigas, D.; McClintock, E.; Drager, N. | 2012 | Negotiation process and context surrounding the Declaration on the TRIPS Agreement and Public Health (Public Health Declaration) in Doha. | Focused on the two negotiating blocs that emerged: the developing country coalition (headed by Brazil, India, and Zimbabwe) and the pro-TRIPS bloc led by the US and including Canada, Japan, Australia, and Switzerland | Access to medicines | Negotiation - global, regional, bilateral |
| Forman, L | 2008 | Pharmaceutical lawsuit and trade challenges against South Africa, which were ultimately dropped in 2001. | South Africa | Access to medicines | Disputes and trade challenges |
| Forman, L | 2012 | Use of HRIA in the Thai-US FTA negotiations in 2006 and its impact on Thailand's position in future negotiations. | Thailand | Access to medicines | National positions taken in negotiations |
| Friel, S.; Baker, P.; Thow, A. M.; Gleeson, D.; Townsend, B.; Schram, A. | 2019 | How the underlying political dimensions of trade agreements in the Australian context enable or constrain coherence between trade and nutrition policy goals. | Australia | Food - nutrition | National level policymaking |
| Hannah, E. N. | 2011 | The European Commissions' negotiating position on TRIPS and access to medicines. | The EU | Access to medicines | Negotiation - global, regional, bilateral |
| Harvey, K. | 2004 | Medicines prices as a negotiation issue in AUSFTA and the political process through which the agreement was reviewed and ultimately amended. | Australia and the USA | Access to medicines | National positions taken in negotiations |
| He, B. G.; Murphy, H. | 2007 | The potential role of NGOs in constructing ‘global social contracts’ focusing on two case studies: the international NGO campaign for an enforceable labour standards clause linked to trade liberalisation at the WTO, and the campaign to clarify the use of the safeguard measures contained in the TRIPS Agreement to increase access to medicines in developing countries. | Global governance and international organisations focus (NGOs) | Access to medicines | Negotiation - global, regional, bilateral |
| Heywood, M | 2001 | Pharmaceutical litigation against South Africa and national dimensions surrounding this. | South Africa | Access to medicines | Disputes and trade challenges |
| Hirono, K.; Haigh, F.; Gleeson, D. Harris, P.; Thow, A. M.; Friel, S. | 2016 | Potential health impacts in Australia of TPP provisions, and the uptake of a HIA influencing policy. | Australia | Access to medicines, tobacco control, alcohol, food - nutrition | National positions taken in negotiations |
| Jandhyala, S. | 2015 | Factors underpinning variations in countries' extent of IP protections, including the presence of domestic IP interest groups (positively associated with IP protections) and domestic public health concerns (negatively associated with IP protections). | Aggregated data from 65 WTO member state countries (table 1 in paper lists countries included in data sample) | Public health general | Domestic implementation |
| Jarman, H. | 2008 | Similarities in the lobbying strategies of NGOs and business actors in the EU trade policymaking context. | The EU | Public health general | Negotiation - global, regional, bilateral |
| Jarman, H. | 2015 | Tobacco trade challenges. | Global | Tobacco control | Disputes and trade challenges |
| Jorge, M. F. | 2010 | How the Government of Peru took advantage of improvements in the US renegotiation of the US-Peru FTA to improve access to medicines. | Focused on actions of Peru | Access to medicines | National level policymaking |
| Koivusalo, M.; Mackintosh, M. | 2011 | The relationship between nongovernment public action and global campaigning on access to medicines and the changing processes of global governance and global health policies. | Global health governance focus | Access to medicines | Negotiation - global, regional, bilateral |
| Krikorian, G. P. | 2017 | The transnational access to medicines movement in Thailand and the 'knowledge activism' that developed in Thailand in response to IP protections in trade negotiations. | Thailand | Access to medicines | National positions taken in negotiations |
| MacNaughton, G.; Forman, L. | 2015 | The context, process, and outcomes of human rights/health impact assessments in Thailand and Peru in negotiations for a bilateral trade agreement with the USA. | Peru and Thailand in bilateral negotiations with the USA | Access to medicines | National positions taken in negotiations |
| Margulis, M. E. | 2018 | UN actors' use of moral and delegated authority to shape global trade rule-making, including the FAO, the World Food Programme, and the UN Special Rapporteur on the Right to Food. | International organisations (UN actors) | Food - security | Negotiation - global, regional, bilateral |
| Moon, S.; Balasubramaniam, T. | 2018 | Policy space for health concerns at the WTO and institutional factors inhibiting or facilitating the protection of health in access to medicines and tobacco control. | General focus on WTO regime | Access to medicines | Negotiation - global, regional, bilateral |
| Morin, J. F.; Gold, E. R. | 2010 | Why policy makers and non-state actors supported the 30^th^ August 2003 Final Decision, despite its significant shortcomings in protecting access to medicines, focusing particularly on the issue of consensus-seeking as a dominant procedural norm in international trade negotiations. | Focused on the WTO level | Access to medicines | Negotiation - global, regional, bilateral |
| Murphy, H. | 2010 | Global – TRIPS and the access to medicines campaign. | Global - WTO | Access to medicines | Negotiation - global, regional, bilateral |
| Nunn, A., da Fonseca, E., Gruskin, S. | 2009 | USA trade disputes against Brazil at the WTO and Doha. | Brazil | Access to medicines | Disputes and trade challenges |
| Odell, J. S.; Sell, S. K. | 2006 | Developing countries’ use of framing as a distributive tactic to elicit gains in the multilateral Doha negotiations and subsequent Declaration on TRIPS and Public Health. | Examined actors on two sides of the TRIPS debate and negotiations: developing countries (Brazil, South Africa, India) and NGOs such as Oxfam and MSF, versus pharmaceutical companies and the developed countries where they are based (The USA). | Access to medicines | Negotiation - global, regional, bilateral |
| Ovett, D. | 2007 | Domestic policy and bilateral trade negotiations, also using global UN body CESCR (Botswana), CRC (El Salvador). | Ecuador, Botswana, Denmark, Italy, also global (UN) | Access to medicines | Negotiation - global, regional, bilateral |
| Owen, T. | 2014 | The ebb and flow of hegemonic (WTO) versus counter-hegemonic (transnational civil society) discourses surrounding IP protections and HIV medicines access. | Global focus | Access to medicines | Negotiation - global, regional, bilateral |
| Rosenberg, S. T. | 2014 | Thailand and Brazil's responses to IP trade rules (TRIPS and TRIPS Plus). | Thailand and Brazil | Access to medicines | NNational positions taken in negotiations |
| Ruff, K. | 2017 | The role of an advocacy campaign involving the scientific community, activists, and asbestos victims in bringing about a ban on the mining, use, and export of asbestos in Canada. | Canada | Asbestos harm | Trade ban |
| Sell, S. K. | 2004 | The structural, discursive, and institutional dimensions of TRIPS and access to medicines from a global governance perspective. | Two groups: those supporting strong IP protections, prominently the US and brand name pharmaceutical companies, versus an alliance of developing country governments (prominently Brazil, India, and the Africa Group) and NGOs | Access to medicines | Negotiation - global, regional, bilateral |
| Sell, S. K.; Prakash, A. | 2004 | How business and NGO groups strategically employ framing to shape the international IP rights regime, focusing on two cases: the success of a transnational network of corporations in grafting their agenda onto the 1994 TRIPS negotiations of the Uruguay Round of GATT, and the success of a transnational NGO network and access to medicines campaign in grafting their agenda onto TRIPS, which resulted in the USA not pursuing IP violations of HIV medicines in developing countries. | Global governance focus – transnational business and NGO coalitions/networks. | Access to medicines | Negotiation - global, regional, bilateral |
| Shadlen, K. | 2004 | The battle over Doha at WTO. | Global | Access to medicines | Negotiation - global, regional, bilateral |
| Thaiprayoon, S.; Smith, R. | 2015 | The development of 'global health diplomacy' capacity in Thailand for trade and health policy coherence, focusing on responses to IP provisions under trade liberalisation (TRIPS and TRIPS-Plus). | Thailand | Access to medicines | National level policymaking |
| T'Hoen, E.; Berger, J.; Calmy, A.; Moon, S. | 2011 | Government and civil society action in developing countries to protect access to HIV medicines in response to the TRIPS Agreement (which culminated in the Doha Declaration). | Developing countries | Access to medicines | Negotiation - global, regional, bilateral |
| t'Hoen, E.F. | 2002 | Multilateral negotiations on IP and access to medicines. | Global | Access to medicines | Negotiation - global, regional, bilateral |
| Thow, A. M.; Annan, R.; Mensah, L.; Chowdhury, S. N. | 2014 | The policy process and outcomes of a domestic food standards policy implemented in Ghana, which limited the amount of fat in meat for import. | Ghana | NCDs | Trade ban |
| Thow, A. M.; Jones, A.; Hawkes, C.; Ali, I.; Labonté, R. | 2018 | Trade concerns regarding interpretive nutrition labelling proposals/policies raised by WTO member states via the TBT Committee. | Specific Trade Concerns related to domestic interpretive nutrition labelling proposals/policies in five countries: Thailand, Chile, Indonesia, Peru, and Ecuador. | Food - nutrition | Trade proofing health policy' - governance conditions |
| Thow, A. M.; Jones, A.; Schneider, C. H.; Labonte, R. | 2019 | Issues surrounding the global governance of front-of-pack (FoP) food labelling, including the potential development of a FoP standard under The Codex Alimentarius, and how decisions about FoP labelling are made and influenced at the global level. | Global governance focus | Food - nutrition | Negotiation - global, regional, bilateral |
| Thow, A. M.; Reeve, E.; Naseri, T.; Martyn, T.; Bollars, C. | 2017 | Agenda-setting, development, implementation, and impact of domestic trade-related policy initiatives implemented in Pacific Island countries to improve the food supply. | Fiji, Samoa, and Tonga | Food - supply | National positions taken in negotiations |
| Thow, A. M.; Swinburn, B.; Colagiuri, S.; Diligolevu, M.; Quested, C.; Vivili, P.; Leeder, S. | 2010 | The removal of Samoa's domestic import policy ban on turkey tails as part of its accession to the WTO, including the rationale for the removal and Samoa's response. | Samoa | Food – supply and nutrition | Trade proofing health policy |
| Townsend, B.; Schram, A.; Baum, F.; Labonté, R.; Friel, S. | 2018 | How actors framed interests to influence the Australian Government during negotiations for the TPP and implications for the incorporation of the social determinants of health in trade policy. | Australia | Public health general | National level policymaking |
| Townsend, B., Friel, S., Schram, A., Baum, FLabonte, R | 2020 | Factors which shaped attention to tobacco control and access to medicines in Australia’s negotiating position in the TPP (in contrast to alcohol control and nutrition). | Australia | Access to medicines, tobacco control | National government positions taken in trade negotiations |
| Townsend, B. | 2021 | Key factors reported by policy actors that shaped the removal of TRIPS Plus IP measures for access to medicines in the RCEP negotiations. | Regional – RCEP negotiations | Access to medicines | Trade negotiations between two or more countries |
| Tuerk, E.; Mashayehki, M. | 2008 | Multilateral, regional, and national trade negotiations on IP and access to medicines and health services. | Asia (Pakistan and the Philippines), Africa (Uganda), and Latin America (Peru) | Public health general, access to medicines | National positions taken in negotiations |
| Voon, T. | 2015 | Issues surrounding empirical evidence requirements to justify domestic public health regulations in international trade and investment law regimes, and key lessons for policy makers and public health advocates to successfully defend trade challenges to public health policy. | Focused on international trade and investment law and arbitration system. Also specific cases - NAFTA case between Canadian company and USA (California) | Public health general | Disputes and trade challenges |
| Vonbraun, J. | 2012 | Political and institutional factors that shaped policy preferences put forward by the Governments of Peru and Columbia in the USA FTA negotiations (particularly in relation to IPR negotiations). | Columbia and Peru | Access to medicines | National positions taken in negotiations |
| Zhou, S. | 2018 | How instruments developed by international health organisations could be used to manage fragmentation between health and international trade and investment law in food and alcohol regulation. | International governance mechanisms focus. Analysis also of trade disputes at WTO and in tribunals | NCDs | Disputes and trade challenges |

WTO: World Trade Organization; TRIPS: Trade-Related Aspects of Intellectual Property Rights Agreement; GATT: General Agreement on Tariffs and Trade; SPS: Sanitary and Phytosanitary Measures Agreement; ASEAN: The Association of Southeast Asian Nations; ISDS: Investor-state dispute settlement; FTA: Free Trade Agreement; HRIA: Human Rights Impact Assessment; HIA: Health Impact Assessment; IP: Intellectual Property; CESCR: Committee on Economic, Social and Cultural Rights; TBT: Technical Barriers to Trade Committee; RCEP: Regional Comprehensive Economic Partnership; IPR: Intellectual Property Rights Agreement.
